# Use of stereotypical mutational motifs to define resolution limits for the ultra-deep resequencing of mitochondrial DNA

**DOI:** 10.1038/ejhg.2014.96

**Published:** 2014-06-04

**Authors:** Kristian Gardner, Brendan AI Payne, Rita Horvath, Patrick F Chinnery

**Affiliations:** 1 Mitochondrial Research Group, Institute of Genetic Medicine, Newcastle University, Newcastle-upon-Tyne, UK

## Abstract

Massively parallel resequencing of mitochondrial DNA (mtDNA) has led to significant advances in the study of heteroplasmic mtDNA variants in health and disease, but confident resolution of very low-level variants (<2% heteroplasmy) remains challenging due to the difficulty in distinguishing signal from noise at this depth. However, it is likely that such variants are precisely those of greatest interest in the study of somatic (acquired) mtDNA mutations. Previous approaches to this issue have included the use of controls such as phage DNA and mtDNA clones, both of which may not accurately recapitulate natural mtDNA. We have therefore explored a novel approach, taking advantage of mtDNA with a known stereotyped mutational motif (nAT>C, from patient with MNGIE, mitochondrial neurogastrointestinal encephalomyopathy) and comparing mutational pattern distribution with healthy mtDNA by ligation-mediated deep resequencing (Applied Biosystems SOLiD). We empirically derived mtDNA-mutant heteroplasmy detection limits, demonstrating that the presence of stereotypical mutational motif could be statistically validated for heteroplasmy thresholds ≥0.22% (*P*=0.034). We therefore provide empirical evidence from biological samples that very low-level mtDNA mutants can be meaningfully resolved by massively parallel resequencing, confirming the utility of the approach for studying somatic mtDNA mutation in health and disease. Our approach could also usefully be employed in other settings to derive platform-specific deep resequencing resolution limits.

## Introduction

Massively parallel (‘next generation') resequencing is potentially a powerful tool with which to study heteroplasmic mitochondrial DNA (mtDNA) mutations because of the possibility of the high breadth and depth of coverage. Studying very low-level variants (below ∼2% heteroplasmy level) is likely to yield the most useful insights into somatic (acquired) mtDNA mutation, but is challenging due to the difficulty in differentiating true variants from background noise at this resolution. Recent studies using amplicon-based resequencing (Roche 454 GS-FLX pyro-sequencing, Branford, CT, USA) have indicated (using mtDNA clones) that mtDNA mutations at ∼0.2% heteroplasmy level or greater can be successfully resolved, but only after very stringent data cleaning, including the exclusion of homopolymeric tracts that are known hot spots for technical artefact on this platform. This approach is therefore not well suited for analysing low-level mutation across whole mtDNA genomes.^1, 2^ In contrast, methods using fragment resequencing (Illumina GA platform, San Diego, CA, USA) have tended to use more conservative limits of resolution (for example of >1.5% heteroplasmy level), which potentially excludes much of the variants of interest.^3, 4^

To further explore this issue, we adopted a novel strategy of ultra-deep resequencing by ligation (Applied Biosystems SOLiD, Foster City, CA, USA) of mtDNA with a known stereotyped mutational pattern and comparing with normal mtDNA. MNGIE (mitochondrial neurogastrointestinal encephalomyopathy) is caused by mutations in the gene *TYMP*, encoding the enzyme thymidine phosphorylase. This nuclear defect results in secondary mtDNA mutations due to purine/pyrimidine pool imbalance. mtDNA point mutations in MNGIE have a very characteristic ‘signature motif' (nAT>C).^5^ We therefore explored the lower heteroplasmy limit to which this stereotypical mutational pattern could be resolved.

## Materials and methods

Total DNA was prepared from skeletal muscle from two patients; one with a confirmed diagnosis of MNGIE (*TYMP* mutation, 22q13.32-qter) and the other from an age-matched healthy subject. Sequencing was performed using the AB SOLiD platform with a 50-bp paired-end fragment library. mtDNA was enriched from total DNA extract by using three overlapping long-range PCR (LR-PCR) fragments, designed to avoid the amplification of nuclear mitochondrial pseudogenes. LR-PCR amplicons were cleaned by column purification and pooled in equimolar amounts per sample.

The raw data were aligned to the revised Cambridge reference sequence (rCRS, NC_012920) using BWA^6^ and processed using SAMtools.^7^ Variant prediction was performed using VarScan.^8^ A bidirectional variant read proportional difference of twofold was permitted between each sequencing strand (single strand biased variants were automatically removed). Putative variants identified were initially categorised by scanning heteroplasmy threshold intervals of 0.1%. VarScan parameters were as follows: minimum coverage, 1500; minimum supporting allele, 10; phred score, 30; variant frequency threshold, 0.1%.

## Results

Mean sequencing read depth across the mtDNA genome was 16 873 and 17 852 for control and MNGIE, respectively. Overall, >95% of the whole mtDNA genome in both samples had read depth of >5000.

**Figure 1:**
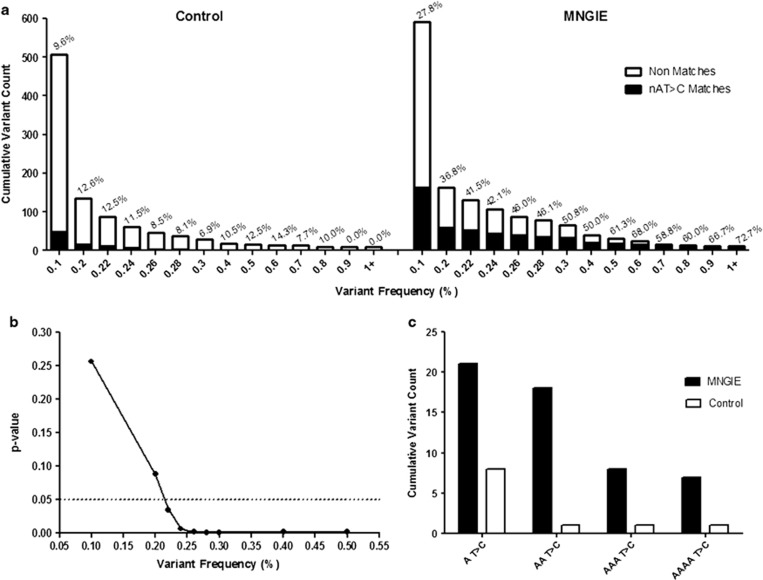
Resolution of low-level heteroplasmic mtDNA variants. (**a**) Cumulative frequency count of detected variants according to variant frequency (heteroplasmy) threshold. Variants are classified according to whether they match the stereotypical motif (nAT>C) or not (nAT>C proportion, as a percentage of all variants detected, is recorded above each threshold). (**b**) Statistical comparison (ANOVA) of mutational spectra (nAT>C motif) between MNGIE and control samples according to variant frequency (heteroplasmy) threshold (dotted line corresponds to *P*-value of 0.05). A significant difference in mutational spectrum could be detected at variant detection thresholds of ≥0.22%. (**c**) Cumulative variants present at ≥0.22% heteroplasmy according to ‘n' value of nAT>C motif.

The expected stereotyped mutational pattern (nAT>C) was seen extensively within the MNGIE mtDNA at all heteroplasmy detection thresholds examined, including the very lowest (0.1%) (Figure 1a). In MNGIE mtDNA, the total number of mutations detected with this stereotyped pattern increased as the variant detection threshold was lowered; however, the proportion of total variants corresponding to the stereotypical pattern progressively increased with increasing detection threshold.

We hypothesised that, at the very lower detection levels, noise (PCR-generated, base-calling and so on) should appear broadly similar across any mtDNA sample both quantitatively (number of ‘variant' calls) and qualitatively (variant pattern). Therefore, to determine the validity of the ‘variants' we detected at the very lowest thresholds (0.1–0.5%), an ANOVA was performed to compare the distribution of stereotyped variants between MNGIE and control mtDNA. In such a manner we determined that, at detection thresholds of ≥0.22%, the excess of nAT>C variants in MNGIE mtDNA is statistically significant (*P*=0.034 for ≥0.22%, Figure 1b), whereas at lower detection levels it is not, suggesting that the stereotypical signal becomes swamped by background noise below 0.22%. The number and distribution of mutations present above this threshold, according to nAT>C motif (where *n*=1–4), is shown (Figure 1c).

Considering the relative proportions of total and stereotypical variants (Figure 1a), we see that at very low heteroplasmy levels (∼0.1–0.2%), the number of non-stereotypical mutations in control and MNGIE mtDNA is very similar, suggesting that any excess of mutations in MNGIE correspond almost entirely to the nAT>C stereotypical pattern. At heteroplasmy thresholds of ≥0.3% we see that in MNGIE mtDNA >50% of all variants correspond to the stereotypical pattern. If we therefore take a very conservative assumption that all non-stereotypical mutations seen at this level are background noise rather than signal, we can conclude that, as a minimum, at heteroplasmy thresholds of ≥0.3% the majority of variants seen will have been generated biologically, rather than as technical artefacts of the sequencing.

## Discussion

The use of a unique biological model based on a specific mutation motif has confirmed that very low-level mtDNA variants (in the <1% heteroplasmy range) can be meaningfully resolved by massively parallel resequencing. We have empirically demonstrated that heteroplasmy detection thresholds in the range ≥0.22–0.3% are appropriate for studies that aim to examine differences in mutational burden between tissues or diseases. These thresholds are strikingly similar to that previously reported (≥0.2%) for short amplicon resequencing^2^ (Roche 454 GS-FLX), but has the distinct advantages of full mtDNA genome coverage (including inclusion of homopolymeric tracts) in the bioinformatic analysis. Furthermore, this approach does not rely on the derivation of heteroplasmy thresholds from cloned or phage DNA, which might be inherently cleaner on deep resequencing. AB SOLiD uses ligation-mediated chemistry and calls bases in pairs; this approach may have theoretical advantages in terms of base-calling fidelity. Our approach could usefully be employed on other platforms to derive platform-specific resolution limits. This study supports the use of massively parallel resequencing to confidently study somatic mtDNA mutations in health and disease.
